# From Susceptibility Loci to Therapeutic Targets: The Genetic Architecture of IgA Nephropathy

**DOI:** 10.3390/ijms27198821

**Published:** 2026-10-02

**Authors:** Mikołaj Dopierała, Nadja Nitz, Jakub Bursy, Antonina Garbowska, Wiktoria Dopierała, Krzysztof Schwermer, Krzysztof Pawlaczyk

**Affiliations:** Department of Nephrology, Transplantology and Internal Medicine, Poznan University of Medical Sciences, 61-701 Poznan, Poland; nadelek01@gmail.com (N.N.); 87273@student.ump.edu.pl (J.B.); 88117@student.ump.edu.pl (A.G.); 88123@student.ump.edu.pl (W.D.); kpawlac@ump.edu.pl (K.P.)

**Keywords:** IgA nephropathy, chronic kidney disease, glomerulonephritis, genetic susceptibility, four-hit hypothesis, genome-wide association study, complement, gene therapy

## Abstract

IgA nephropathy is the most common primary glomerulonephritis worldwide and a leading cause of chronic kidney disease and kidney failure in young and middle-aged adults, characterized by considerable ethnic differences in incidence and prognosis. This review summarises the evidence that these differences are, at least in part, genetically determined and maps the identified susceptibility loci onto the pathogenic sequence of the disease. Worldwide prevalence gradient increases with eastward and northward distance from Africa, while showing the significance of ancestry rather than environment in diaspora populations. Familial clustering provides support for an inherited component, while methylation differences between monozygotic twins discordant for IgAN point to an additional epigenetic layer. Genome-wide association studies (GWAS) in Han Chinese, European, and Korean cohorts have identified susceptibility loci implicated in antigen presentation, mucosal and intestinal IgA immunity, complement regulation, and innate immune signaling. Mapping these loci onto the four-hit hypothesis shows that inherited variation acts at distinct, potentially separable nodes. The MHC region contributes approximately half of the SNP-based heritability, while the *CFH/CFHR* locus provides the strongest signal outside the MHC. The complement arm has become the principal therapeutic target because it is currently the most pharmacologically tractable. We conclude by reviewing mechanism-directed therapies together with the safety questions and patient-selection issues that accompany them.

## 1. Introduction

Chronic kidney disease (CKD) is a major and growing global health problem. In 2023, it affected an estimated 788 million adults worldwide, more than twice as many as in 1990, and it was the ninth leading cause of death, accounting for approximately 1.5 million deaths. Although diabetes and hypertension account for most of this burden, glomerular diseases remain an important cause of CKD in younger patients, in whom the loss of kidney function extends over decades of working life. IgA nephropathy (IgAN) is the most common primary glomerulonephritis worldwide and an important cause of kidney failure in young and middle-aged adults, in whom a substantial proportion of patients progress to kidney failure within two decades of diagnosis. Because the disease typically begins in early adulthood, most patients are at risk of kidney failure within their expected lifetime [1,2,3]. Diagnosis still rests on the demonstration of dominant or codominant mesangial IgA deposits on renal biopsy, and management has, until very recently, consisted of generalized chronic kidney disease care with immunosuppression added empirically rather than on mechanistic grounds. Current guidelines aim to reduce proteinuria below 0.5 g/day, ideally below 0.3 g/day, and to slow kidney function loss to less than 1 mL/min/1.73 m^2^ per year [4].

That the disease is not distributed evenly across the world has been recognized for decades. IgAN is diagnosed far more often in East Asian and European populations than in populations of African ancestry, and biopsy-based series show an approximately ninefold difference in the proportion of biopsies attributable to IgAN between East Asia and Africa [1,2,3]. Such observations are open to more than one explanation: differences in biopsy policy, in access to nephrological care, in the prevalence of mucosal infections and in diet could all generate an apparent gradient without any underlying difference in susceptibility. Disentangling ascertainment and environment from inherited risk has therefore been a central problem in the epidemiology of IgAN, and one that genetic data can potentially resolve.

In this review, we summarise the epidemiological, familial, twin-based, and genome-wide evidence for a genetic contribution to IgAN, with attention to differences and similarities between populations. We then map the implicated genes and variants onto the four-hit hypothesis, integrating genetic regulation with epigenetic regulation, where the two converge on the same molecular target. Finally, we review the therapies that follow directly from this molecular understanding and discuss the safety considerations and patient selection questions that will determine whether they can be translated into routine practice.

## 2. Methods

Literature used in the review was identified primarily through PubMed. Large language model-based assistants were used as supportive tools to help identify recently published articles (ChatGPT, Calude Opus). The search concentrated on the newest publications. Reference lists of retrieved articles were screened for additional sources. We included original studies, meta-analyses, and guidelines addressing the genetic, epigenetic, or molecular basis of primary IgAN and mechanism-directed therapy. We also excluded papers on secondary IgA deposition or IgA vasculitis nephritis not relevant to primary IgAN, as well as case reports without mechanistic insight. Publications that have been superseded by a larger or more recent analysis of the same cohort were also dismissed. We prioritized large, replicated GWAS and studies from the last five years and retained older work when it was the primary source of a concept.

## 3. Geographic, Ethnic, and Racial Variation

IgA nephropathy (IgAN) is a common kidney disease, particularly among European and Asian populations, where it is more frequently diagnosed than among people from Africa and the Americas. The frequency of the disease is highest in East Asia, accounting for nearly 45% of patients who undergo renal biopsies. The prevalence of IgAN is moderate in European populations, at around 25% of renal biopsy results, while African populations have the lowest rate at just under 5% [1,2]. In the USA, it is difficult to draw conclusions regarding this issue due to the diversity of the population. The reported incidence rate of IgAN ranged from 0.39 per 100,000 in Tennessee to 1.4 per 100,000 in Minnesota [2].

The geographic variation parallels the distribution of genetic risk. Evidence comes from a genome-wide association study (GWAS) replication and geospatial risk analysis by Kiryluk et al. [3]. The authors created a genetic risk score based on seven validated susceptibility loci and applied it across 85 world populations. The score was highest in East Asian populations, intermediate in Europeans, and lowest in Africans, closely paralleling the known worldwide distribution of IgAN prevalence. The risk score increased steadily with both eastward and northward distance from Africa, and it predicted a previously unrecognized North–South gradient within Europe—a prediction subsequently confirmed by registry data showing higher rates of IgAN-attributable kidney failure in Northern European countries, mirroring the latitudinal patterns observed in other immune-mediated diseases like multiple sclerosis and type 1 diabetes. Importantly, the same covariation between genetic risk and IgAN-related kidney failure was observed among Asian Americans, European Americans, and African Americans, who share a genetic background but not an environment with their ancestors. This observation shows that the gradient may be driven by inherited susceptibility and not by geography or environment alone. The analysis delivers the integrative link between the epidemiological observation that disease frequency differs between populations and the conclusion that these differences are, at least in part, genetically determined [3]. These analyses are nonetheless ecological: they compare aggregate risk scores with population-level disease frequencies and cannot separate genetic ancestry from environmental, dietary and infectious exposures that vary along the same geographic axes, nor from population structure itself.

A study conducted at the University of California, San Francisco, in 1994–2001 involved 298 patients with primary glomerular disease—149 with IgAN and a random sample of 149 with non-IgAN glomerulonephritis. Among patients with IgAN, the cohort included a substantial proportion of White and Asian/Pacific Islander patients. Clinical parameters like proteinuria and the presence of hypertension, as well as histopathologic severity (Haas subclass), showed no significant difference across major racial/ethnic groups. One noticeable finding was that African American patients had higher serum creatinine levels than other groups. Overall, the study suggested differences in the racial/ethnic distribution of IgAN compared with other primary glomerulopathies, but did not demonstrate a clear association between race/ethnicity and IgAN severity among biopsied patients [5,6].

Another study from the Toronto Glomerulonephritis Registry included 202 patients with IgAN of Pacific Asian origin and 467 patients of other backgrounds. After adjusting for age, sex, baseline kidney function, blood pressure, proteinuria and treatment-related variables, the authors found that Pacific Asian origin was associated with a higher risk of progression to kidney failure. Contrary to the previously cited study, here the results suggest the existence of ethnic differences in terms of prognostic significance for patients with IgAN [7].

## 4. Familial Aggregation and Inheritance

A number of studies have shown familial aggregation of IgAN [8,9,10]. In the majority of cases, the most likely pattern of genetic inheritance is autosomal dominant with variable penetrance, but other genetic models are also possible [11]. The familial variant of the disease is more common in families of European origin, as demonstrated by several studies [9,10,12,13].

The incidence of IgAN also differs by sex. In men, it is five times higher than in women, with the male gender being a predictor of poor renal outcomes [14]. In addition, familial cases are characterized by poorer renal outcomes and a lesser sex difference than in sporadic variants [9,15].

In a genome-wide analysis of 24 IgAN families from Italy and six families from the USA, a susceptibility locus on chromosome 6q22-23 was identified, which could be linked to autosomal dominant inheritance with incomplete penetrance [16]. Another study conducted on a Sicilian family discovered a variant of the *SPRY2* gene that can inhibit the mitogen-activated protein kinase/extracellular signal-regulated kinase 1/2 (MAPK/ERK1/2) pathway, which is responsible for the growth, proliferation, and apoptosis of cells. This variant is linked to the appearance of the dominant variant of IgAN [10,17,18]. A study with participants from one family of German–Austrian origin found mutations on chromosome 2q36. The mutation increases susceptibility to the disease and was identified in 14 family members with IgAN [13]. Another study of 10 Irish families with IgAN revealed two mutations in genes encoding type 4 collagen, which can cause deposition of vascular-derived IgA molecules in the mesangial area of the glomeruli [12].

Several clues suggest the contribution of *COL4A3/COL4A4/COL4A5* variants to familial IgAN. Locus 2q36, on which the *COL4A3* and *COL4A4* genes are located, was identified as a susceptibility locus for familial IgAN [13]. Pathogenic *COL4A3/COL4A4/COL4A5* mutations were detected in approximately 20% of patients with familial IgAN [19]. It cannot be excluded that some of these families represent autosomal dominant collagen IV-related nephropathy with coincidental mesangial IgA deposition rather than familial IgAN with a collagen IV modifier.

## 5. Twin Studies and Heritability

A study by Lomax-Browne et al. investigated 148 healthy female twins (47 pairs of dizygotic twins and 27 pairs of monozygotic twins) from the UK. The researchers measured serum levels of Gd-IgA1 and total IgA. The investigators showed that (galactose-deficient IgA1) Gd-IgA1 levels are highly heritable (heritability ~80%), indicating a stronger genetic than environmental contribution to this trait [20].

Wei et al. investigated genome-wide DNA methylation patterns in monozygotic twins in which only one twin developed IgAN. The study demonstrated that differences in DNA methylation were associated with IgAN pathogenesis, independent of genetic background. Functional enrichment analysis identified multiple differentially methylated genes involved in inflammatory and immune response pathways, which further supports the role of epigenetic regulation in IgAN development [21].

## 6. Genetic Architecture

IgAN is highly polygenic. SNP-based heritability has been estimated at 0.23, falling to 0.12 when the MHC region is excluded, indicating that HLA and non-HLA loci contribute approximately equally to polygenic risk and that the MHC alone accounts for about half of the estimated heritability. A risk score based on the 30 genome-wide significant variants explains approximately 11% of disease variance, compared with 6% for the earlier 15-SNP score, and a genome-wide polygenic score explained about 7.3% of disease risk in an independent cohort. Most of the heritability remains unaccounted for by currently identified loci. Higher polygenic burden was associated with earlier diagnosis and with faster progression to kidney failure, although the clinical utility of such stratification has not been established prospectively.

Within the MHC, the association does not arise from a single variant. At least five variants in the region are independently associated with IgAN. Fine mapping pointed to class II genes, with *DRB1* most strongly implicated in both European and East Asian cohorts. The authors concluded that extended linkage disequilibrium across the region, population differences in haplotype diversity, and the limitations of imputation prevent attribution of these signals to a single causal gene. The MHC therefore carries the largest genetic contribution in IgAN, but it is not resolvable to specific genes within the region.

## 7. GWAS and Candidate Genes

GWAS provide direct evidence for a contributory role of genetics in IgAN by identifying risk and protective loci and showcasing the similarities and differences between populations.

One such study was performed on the Chinese Han ethnic group by Yu et al. In the first stage, they studied 1434 affected individuals and 4270 controls and followed up with the top 61 single-nucleotide polymorphisms (SNPs) in an additional 2703 cases and 3464 controls. They identified associations that implicated the genes encoding APRIL—a proliferation-inducing ligand (a TNF-superfamily ligand, encoded by *TNFSF13*) and α-defensin (encoded by *DEFA*) as susceptibility genes, and found multiple associations in the major histocompatibility complex (MHC) region. What is more, the authors concluded that rs660895 was connected to clinical subtypes of IgAN, proteinuria, and IgA levels [22].

Testing for IgAN in the Han ethnic group using an exon chip uncovered novel variants of suggestive significance in the human leukocyte antigen (HLA) region and in the *FBXL21*, *CCR6*, and *STAT3* regions, which are involved in the biological pathways of immunomodulation of IgA production and complement activation [23]. Risk allele frequencies tend to be higher in this Chinese population than in Europeans [24]. A study by Li et al. on this population in October 2023 also identified two novel IgAN risk variants, i.e., *VEGFA* and *PKD1L3*. Both are nonsynonymous coding variants and the presence of the *VEGFA* gene variant was associated with a twofold increase in the risk of developing the disease [25].

The rs6677604(A) allele of *CFH/CFHR1-CFHR3* tags a deletion of the Complement Factor H-Related Protein 1 (CFHR1) and Complement Factor H-Related Protein 3 (CFHR3) genes and has been shown to act as a protective allele against the development of IgAN. It has been demonstrated to be more prevalent in the European population than in the Chinese population [3,26,27].

The rs4833095 and rs5743557 polymorphisms in *TLR1* (toll-like receptor 1 encoding gene) have been associated with increased IgAN risk in both Chinese Han adults [28] and Korean pediatric patients [29]. A meta-analysis conducted by Ding et al. confirmed these findings across both populations, additionally demonstrating that the rs4833095-T/rs5743557-T haplotype is a risk factor for IgAN [30]. Notably, the allele distribution of rs4833095 differs between ethnic groups: the T allele—associated with increased IgAN risk—is the major (more common) allele in Europeans, whereas the C allele predominates in Asian and African populations. Despite the T allele being less common in Asian populations, IgAN prevalence remains significantly higher in Asia, showing that additional genetic and/or environmental factors may account for the demonstrated disparities in disease frequency between different ethnic groups [30].

SNPs rs12054151, rs2239611, rs1990677, rs4686838, rs2284750, rs6784233 and rs7634389 in Beta-galactoside alpha-2,6-sialyltransferase 1 (*ST6GAL1*) have been linked with IgAN incidence. The association at rs7634389 reached genome-wide significance in a multi-stage GWAS of Han Chinese patients. Two studies included Chinese Han and Chinese Uyghur populations and showed that rs3840858 in ST6 N-acetylgalactosaminide alpha-2,6-sialyltransferase 2 (*ST6GALNAC2*) is associated with susceptibility to IgAN [30]. Variants of these two molecules are not only related to the risk of developing IgAN, but also to its severity [31].

The SNP rs4151657 polymorphism in the complement factor B (*CFB*) gene showed a stable association with IgAN risk in the Han Chinese population (in dominant allele, superdominant, and heterozygous models). It was also associated with higher serum CFB levels and reduced serum C3 levels, indicating poorer renal outcomes [32,33]. It has been observed that the minor A allele of the *TNFSF13* gene (rs3803800) shows a significant correlation with IgAN in additive and recessive models and is linked to several clinical phenotypes: severe proteinuria, low estimated glomerular filtration rate (eGFR), and a higher prevalence of mesangial hypercellularity [34].

Regarding inflammatory cytokine genes, a meta-analysis of immune and inflammatory gene polymorphisms demonstrated that rs1946518 in *IL18* is associated with increased IgAN susceptibility in both Chinese Han and Korean populations [30].

Finally, genetic variants of *COL4A3/COL4A4/COL4A5* were found in a subset of patients with sporadic IgAN, where they were associated with thinned glomerular basement membrane lesions, suggesting a potential contribution of collagen IV pathway dysregulation to IgAN pathology, although these variants may equally indicate a coexisting or alternative collagen IV-related nephropathy [35].

GWAS performed on European and Chinese populations identified multiple susceptibility loci. Most were similar for both populations [24,36]. A meta-analysis by Chronopoulou et al. showed that the presence of genetic variants and haplotypes in the *IL1B, IL1RN* and *IL10* genes may increase the risk of IgAN development and progression in populations of European ancestry [37]. Hahn et al. demonstrated an association between the presence of the *IL1B* and *IL1RN* variants and susceptibility to IgAN in children, as well as between *IL1A* and the onset of proteinuria [38]. Bantis et al. demonstrated the effect of the C-344T polymorphism of the aldosterone synthase gene (*CYP11B2*), which is associated with serum aldosterone levels and the risk of hypertension, on disease progression in patients of European ancestry with IgAN, suggesting a role for aldosterone-mediated pathways in IgAN [39]. These findings come from single-ancestry candidate-gene studies and meta-analyses of such studies; none of these loci reached genome-wide significance in the multi-ancestry GWAS.

In a study performed on the Korean population, a SNP in the *ANKRD16* locus (ankyrin repeat domain 16) showed a nominal association with IgAN [40]. ANKRD16 is a vertebrate-specific protein that plays a crucial role in maintaining translational fidelity and mitigating the consequences of defective transfer RNA (tRNA) synthetase editing [41]. The study had a small sample size, so the results require validation in future studies, but it suggests a role of this variant in the appearance of IgAN on the Korean peninsula [40]. The key evidence for a genetic contribution to IgAN is presented in Table 1.

## 8. The Four-Hit Hypothesis

The 4-hit hypothesis describes the most widely accepted model for the steps that cause progressive kidney damage in IgAN. The pathway begins with increased production of Gd-IgA1. This is known as the first hit. The second hit constitutes the formation of autoantibodies against Gd-IgA1. Next comes the creation of circulating immune complexes containing Gd-IgA1 and the aforementioned autoantibodies—the third hit. The final, fourth hit describes the deposition of those pathogenic immune complexes in the kidneys, which in turn leads to inflammation and kidney injury. The kidney damage itself is not included in the 4-hit hypothesis, but can be treated as the next, natural step of the process. Each step of the mentioned pathophysiological process can vary and be modified in correlation with different genetic patterns in individual patients.

### 8.1. The First Hit

The aberrant O-glycosylation of the IgA1 hinge region, that results in elevated levels of circulating Gd-IgA1 is governed by an enzymatic cascade in which three genes play central roles: *C1GALT1*—encoding core 1 β1,3-galactosyltransferase, which is responsible for adding galactose to N-Acetylgalactosamine (GalNAc), *C1GALT1C1*—encoding its chaperone Cosmc (core 1 β3-Gal-T-specific molecular chaperone), and is essential for C1GalT1 stability and function, and *ST6GALNAC2*—encoding ST6GalNAc-II, which adds sialic acid to GalNAc residues [42].

Under normal conditions, galactosylation by C1GalT1 precedes sialylation; however, in IgAN patients, overexpression of ST6GalNAc-II results in premature α2,6-sialylation of GalNAc, which sterically blocks subsequent galactosylation by C1GalT1. That process leads to the increased production of Gd-IgA1 [42,43]. The overexpression of ST6GalNAc-II has been attributed to a convergence of multiple regulatory mechanisms. The three main mechanisms are: the influence of cytokines, particularly interleukin-6 (IL-6), which simultaneously upregulates *ST6GALNAC2* and downregulates *C1GALT1*; the presence of genetic variants in the *ST6GALNAC2* promoter region [31]; and epigenetic dysregulation via microRNAs (miRNAs) [44].

Case–control studies demonstrated that SNPs in *C1GALT1* and *ST6GALNAC2* are associated with IgAN susceptibility, and that haplotype combinations across these two genes interact to influence both Gd-IgA1 levels and disease severity [31]. As previously mentioned, family-based studies suggest that serum levels of Gd-IgA1 are heritable in 40–80% of cases [20].

Innate immune signaling represents an additional upstream trigger of the glycosylation defect. *TLR1*, encoding Toll-like receptor 1, is located on chromosome 4p14. TLR1 forms heterodimers with Toll-like receptor 2 (TLR2) and recognizes bacterial lipoproteins at mucosal surfaces, leading to nuclear factor κB (NF-κB) activation and cytokine release, including IL-6, which, as was described before, simultaneously upregulates *ST6GALNAC2* and suppresses *C1GALT1*, linking innate immune mucosal activation to aberrant IgA1 glycosylation. As mentioned in the first chapter, the risk variants rs4833095 and rs5743557 in *TLR1* have been associated with increased IgAN susceptibility in candidate-gene studies in both Han Chinese adults and Korean pediatric patients. In patients in whom these variants occur together as a haplotype, the risk is escalated further [28,29,30].

Two subsequent GWAS for serum Gd-IgA1 levels identified genome-wide significant signals at *C1GALT1* (rs13226913) and *C1GALT1C1* (rs5910940), which jointly explain approximately 7% of Gd-IgA1 variability in Europeans and 2% of Gd-IgA1 variability in East Asians [45,46]. The allele of rs13226913, which increases Gd-IgA1 levels, is common in Europeans but rare in East Asians, which is consistent with higher observed Gd-IgA1 levels in patients of European ancestry and may partly contribute to differences in disease phenotype between these populations. A more recent GWAS in a Chinese cohort identified a novel locus at *GALNT12* (a GalNAc-transferase that initiates O-glycosylation), which showed a genetic interaction with *C1GALT1* in both Gd-IgA1 levels and IgAN susceptibility [23].

Alongside genetic variation, epigenetic regulation is an additional layer of control over the glycosylation process and its potential pathology, as another mechanism contributing to the first hit of IgAN. Epigenetic mechanisms, unlike DNA sequence variants, are acquired and potentially reversible alterations that modify gene expression without changing the underlying genetic code.

Hypermethylation of the *C1GALT1* promoter region has been connected to reduced transcription of the gene, which in turn leads to reduced C1GalT1 protein levels and consequently impaired galactosylation of IgA1 [44].

MicroRNAs (miRNAs) represent a second epigenetic mechanism. Mononuclear cells in the peripheral blood of IgAN patients are characterized by an upregulated miR-148b, which directly targets the 3′UTR (untranslated region) of *C1GALT1* mRNA, reducing its expression at the post-transcriptional level. Importantly, miR-148b levels positively correlate with serum Gd-IgA1 concentrations. Moreover, experimental transfection of healthy B cells with miR-148b mimics was sufficient to increase Gd-IgA1 production [44].

A second miRNA, let-7b, targets *GALNT2*—the gene encoding the enzyme responsible for the first step of IgA1 O-glycosylation, namely the attachment of GalNAc to serine and threonine residues in the hinge region. Since C1GalT1 can only add galactose to GalNAc that has already been attached, downregulation of *GALNT2* by let-7b impairs the entire glycosylation cascade at its earliest step [47].

These findings indicate that the glycosylation defect underlying the first hit reflects a convergence of genetic predisposition and acquired epigenetic alterations.

### 8.2. The Second Hit

The second hit is the production of autoantibodies directed against the exposed GalNAc residues of Gd-IgA1. Elevated circulating Gd-IgA1 alone is insufficient to cause glomerular injury. The critical additional event is the recognition of terminal GalNAc as a neoepitope by glycan-specific autoantibodies, predominantly of the IgG isotype [48].

The genetic basis of the second hit is less understood and researched than that of the first. As mentioned before, the MHC region on chromosome 6p21 is the strongest susceptibility locus in IgAN and accounts for approximately half of the estimated polygenic risk [49]. Specific MHC class II alleles within this locus are hypothesized to act as permissive factors for the second hit development. By presenting the Gd-IgA1-derived glycopeptides to CD4+ T helper cells, these MHC variants have been linked to a T cell-dependent immune response that drives production of anti-glycan IgG autoantibodies. Individuals lacking such permissive alleles may present elevated Gd-IgA1 without a pathogenic autoantibody response. That is why we can reason that elevated Gd-IgA1 alone is not sufficient to cause disease [3,26].

A GWAS locus on chromosome 17p13 implicated the proliferation-inducing ligand (APRIL), which is encoded by *TNFSF13*. Its receptor, transmembrane activator and CAML interactor (TACI), is encoded by *TNFRSF13B* on chromosome 17p11.2. This second gene was identified as an independent risk locus in the multi-ancestry GWAS. Both members of this ligand–receptor pair are therefore supported by genome-wide significant associations and may contribute to the second hit [49].

APRIL is produced primarily by monocytes and macrophages and binds to TACI and related receptors on B-cells, promoting their survival, driving class switching to IgA, and stimulating antibody production.

The risk variant rs3803800 in *TNFSF13* is a missense variant and one of only two lead signals in the multi-ancestry GWAS that map to a coding region [49]. It may affect APRIL activity by altering its post-translational processing; the precise functional consequences of this variant are not yet completely characterized.

The functional consequences of the risk alleles at *TNFRSF13B* have not been defined. A mechanism of this kind has been demonstrated for a related gene, *TNFSF13B*, which encodes BAFF. An insertion–deletion variant in this gene generates a shortened transcript that escapes inhibition by miRNA. This raises levels of soluble BAFF and of circulating immunoglobulins, and increases the risk of multiple sclerosis and systemic lupus erythematosus. Whether a comparable mechanism operates at *TNFRSF13B* in IgAN has not been tested [50].

IgG autoantibodies from IgAN patients possess a characteristic amino-acid substitution in the antigen-binding region of their heavy chain. This change greatly improves binding to Gd-IgA1. Importantly, the substitution arises through a somatic mutation during an active immune response and is not an inherited genetic variant [51].

### 8.3. The Third Hit

The third hit involves the formation of circulating immune complexes between Gd-IgA1 and the anti-glycan autoantibodies produced during the previous hit; it is a physicochemical reaction and lacks defined, independent genetic determinants [52,53].

The resulting immune complexes are large—exceeding 800 kDa—and their size is central to their pathogenicity. Normally, monomeric IgA1 is cleared by hepatocytes via the asialoglycoprotein receptor, which recognizes terminal GalNAc residues. However, once incorporated into a large immune complex, the GalNAc epitopes are blocked by the bound autoantibody, and the size of the complex prevents passage through the narrow fenestrae of the hepatic sinusoidal endothelium. The complexes, therefore, persist in the circulation and deposit in the mesangium via the larger fenestrae of glomerular capillaries [54,55].

Notably, polymeric Gd-IgA1 can also self-aggregate via intermolecular disulfide bonds, forming nephritogenic complexes independently of anti-glycan autoantibodies [53,56].

### 8.4. The Fourth Hit

The fourth hit describes the deposition of immune complexes containing Gd-IgA1 in the glomerular mesangium. In the original hypothesis, the subsequent inflammatory response and glomerular injury are treated as consequences of the fourth hit rather than as a separate step. For clarity, we discuss complement activation and the resulting kidney damage together with the fourth hit throughout this review. The immune complexes activate resident mesangial cells, stimulate their proliferation, promote extracellular matrix expansion, and induce the secretion of pro-inflammatory mediators. These changes are graded histologically by the Oxford MEST-C classification (mesangial and endocapillary hypercellularity, segmental sclerosis, interstitial fibrosis/tubular atrophy and crescents), which is the standard measure of injury severity in IgAN.

These pro-inflammatory molecules recruit circulating monocytes, damage glomerular endothelial cells, and injure podocytes [57].

Complement activation through both the alternative and the lectin pathways is a central amplifier of the fourth hit and the step with the clearest genetic modulators [58].

While the pathophysiological sequence is largely shared across patients, the severity and susceptibility to the fourth hit are modulated by inherited genetic variation.

The principal genetic determinant of the fourth hit is the *CFH/CFHR* locus on chromosome 1q32, which encompasses the complement regulatory genes *CFHR1* and *CFHR3*, influencing the susceptibility to IgAN through the modulation of the alternative complement pathway. Factor H (FH), encoded by the neighboring *CFH* gene, is the main inhibitor of the alternative complement pathway. *CFHR1* and *CFHR3* encode structurally similar proteins that compete with FH for C3b binding but lack its full inhibitory activity. That is why their presence partially antagonizes FH and promotes complement activation [27]. A common 84 kb deletion removing both *CFHR3* and *CFHR1* (*CFHR3,1Δ*) is almost always co-inherited with the protective allele rs6677604-A. Therefore, rs6677604-A serves as a genetic marker for the deletion, given its near-complete co-inheritance (linkage disequilibrium). This haplotype enhances inhibition of the alternative complement pathway mediated by FH, which is associated with lower mesangial C3 deposition and leads to an approximate 40% reduction in IgAN risk per copy [26,27,59]. The frequency of *CFHR3,1Δ* varies across populations: being present in ~20–27% in Europeans to ~7% in Han Chinese and near-absent in some East Asian groups. These differences may partially explain the higher susceptibility to complement-mediated glomerular injury observed in Asian populations. This locus is the one association in IgAN for which the chain from locus to gene has been completed. In two independent Han Chinese cohorts, the deletion itself was genotyped directly rather than inferred from a tagging variant. It accounted for the entire association signal at the locus: the deletion remained significant after the effect of rs6677604 was removed from the model, and no other causal variant was found within the *CFH* cluster [27]. Elsewhere in IgAN, genes are assigned to loci mainly on the basis of proximity to the lead variant, supported by functional annotation. The *CFH*/*CFHR* locus therefore serves as a benchmark for the strength of genetic support behind the therapeutic targets discussed in Section 9.

An additional genetic modulator of complement activation in the fourth hit is *CFB*. The risk variant rs4151657 in the *CFB* gene has been associated with higher serum CFB levels and reduced serum C3 levels in Han Chinese patients, consistent with more pronounced complement-mediated glomerular injury [33].

Genetic variants occurring in the type IV collagen genes *COL4A3*, *COL4A4* and *COL4A5*, which encode the structural framework of the glomerular basement membrane (GBM), have been reported in patients with sporadic IgAN and thinned GBM lesions. Patients carrying diagnostic variants had milder glomerular injury and lower Gd-IgA1 levels than patients with thinned GBM but without such variants, which led the authors to propose that the two groups represent different mechanisms [35]. These may indicate: a collagen IV-related nephropathy in which mesangial IgA deposition is incidental, a diagnostic overlap between the two entities, or a dual pathology, rather than susceptibility to IgAN itself. These variants are therefore discussed here as a differential-diagnostic consideration rather than as part of the genetic architecture of the four hits.

In familial IgAN, pathogenic variants in these genes are detected in approximately 20% of cases, and the *COL4A3/COL4A4* locus on chromosome 2q36 has been identified as a familial susceptibility locus [13,19].

The most comprehensive genetic characterization of IgAN comes from a large multi-ancestry GWAS, which identified 30 genome-wide significant risk loci (named here after the candidate gene prioritised in the source publication—see Section 10), where 16 of them were newly discovered [49]. Pathway analysis of these loci revealed that the implicated genes cluster around four biological processes: antigen presentation (MHC region), mucosal immunity and intestinal IgA production (*TNFSF13*, *DEFA*, *LIF/OSM* loci), alternative complement pathway regulation (*CFH/CFHR* locus), and innate immune signaling via the NF-κB pathway (*RELA*, *REL*, *LYN* loci). These outcomes indicate that susceptibility to the fourth hit and to IgAN as a whole is not determined by a single pathway. Variants across multiple immune and inflammatory pathways interact to define the individual risk of each patient.

Mapping the susceptibility loci onto the four-hit sequence reveals that IgAN is a stepwise process in which inherited variation acts at distinct, and potentially separable, points: glycosylation-enzyme genes are associated with the level of circulating Gd-IgA1, MHC class II alleles and variants in genes encoding APRIL-TACI have been linked to an autoantibody response mounted against it, and the *CFH/CFHR* and *CFB* loci correlate with the intensity of complement-mediated injury once immune complexes have deposited.

It should be emphasized that confidence in an association and confidence in its placement within the four-hit sequence are two different things, and that the two may diverge considerably across the loci discussed above.

For the MHC, the association is the strongest in the disease, yet the assignment is weak: the region harbors at least five independent signals that cannot be resolved to individual class II genes. Furthermore, its placement at the second hit rests on the immunological plausibility of antigen presentation to T helper cells rather than on direct evidence that these alleles act at this step.

For the APRIL-TACI axis, the assignment is better supported. *TNFSF13* and *TNFRSF13B* are independent genome-wide significant loci encoding a ligand–receptor pair.

For the complement loci, the evidence is the most complete. A causal allele has been identified, and its effects at the protein level have been demonstrated. Even here, however, the allocation of *CFHR1* and *CFHR3* to the fourth rather than the third hit reflects the structure of the model rather than an experimental finding, and the third hit has no identified genetic determinant of its own.

Loci implicated through pathway annotation alone, including *DEFA*, *LIF/OSM* and the NF-κB genes *RELA*, *REL* and *LYN*, are placed by inference from the biological processes in which their candidate genes participate. Most of them plausibly act at more than one step.

Each of these nodes is a potential therapeutic target. The complement arm has become the focus of the therapies discussed below because it is the node at which a causal allele, a measurable pathway and available inhibitors coincide.

The genetic contributions to each of the four hits are presented in Table 2.

**Figure 1:**
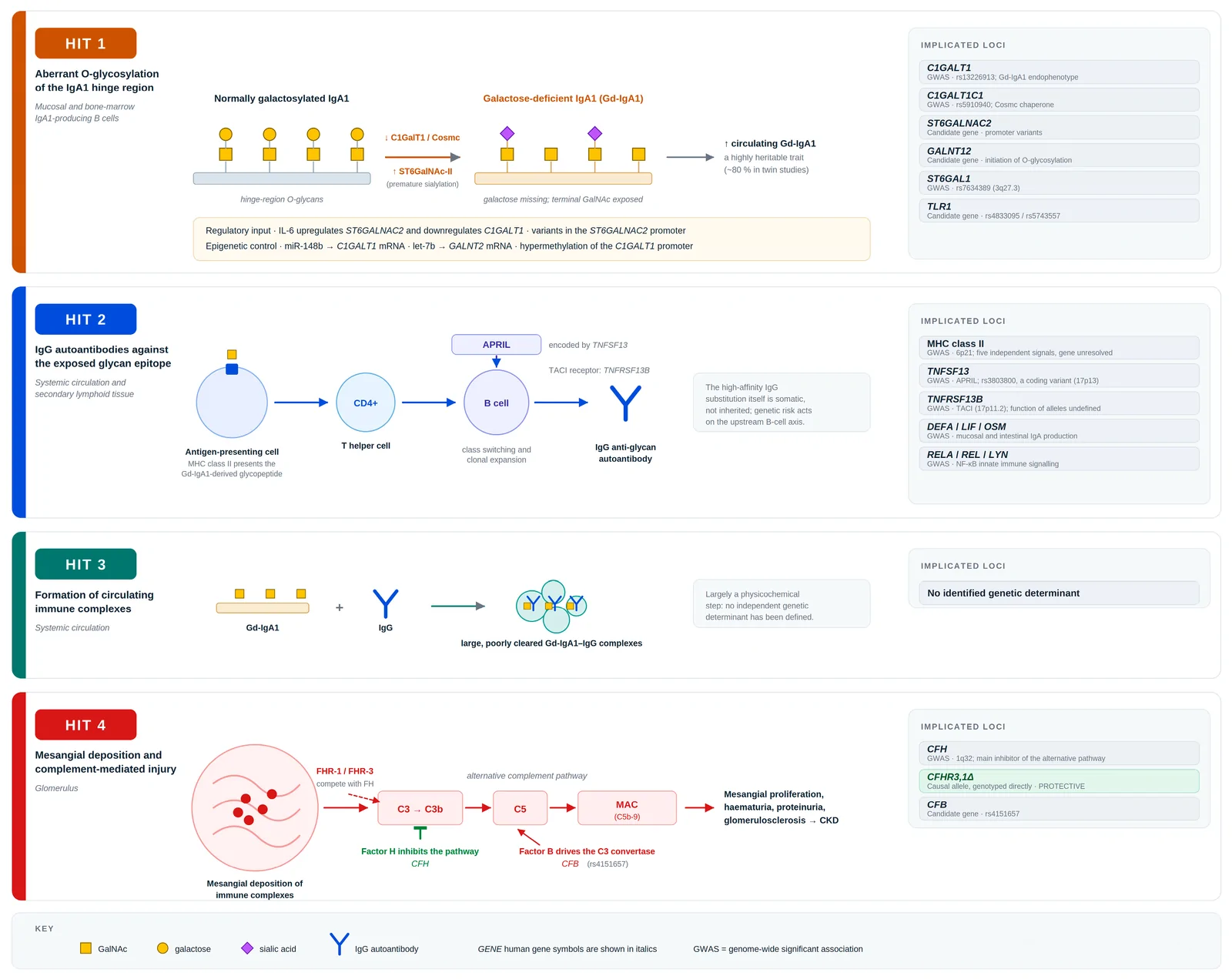
Four-hit hypothesis and proposed genetic contributions. Panels follow the four hits in order; the accent colour identifies the step and carries no quantitative meaning. Arrows show the direction of the sequence, except in the fourth hit, where the dashed arrow marks competition for C3b binding and the blunt-ended connector marks inhibition; there, green denotes protective or inhibitory elements, including the shaded *CFHR3,1Δ* entry, and red denotes activating ones. Upward and downward arrows denote increased and decreased activity. Symbols are defined in the key at the foot of the figure, human gene symbols are italicised and protein names roman, and "GWAS" and "candidate gene" indicate the level of genetic evidence for each locus. Abbreviations: APRIL, a proliferation-inducing ligand; C1GalT1, core 1 β1,3-galactosyltransferase; C3b, activated fragment of complement component 3; CKD, chronic kidney disease; Cosmc, core 1 β3-Gal-T-specific molecular chaperone; FH, complement factor H; FHR-1/FHR-3, complement factor H-related proteins 1 and 3; GalNAc, N-acetylgalactosamine; Gd-IgA1, galactose-deficient IgA1; GWAS, genome-wide association study; MAC, membrane attack complex; MHC, major histocompatibility complex; ST6GalNAc-II, ST6 N-acetylgalactosaminide α-2,6-sialyltransferase 2; TACI, transmembrane activator and CAML interactor.

The four-hit hypothesis with proposed genetic contributions is shown in Figure 1.

The following section reviews these approaches, from gene addition therapy to nucleic acid-based complement inhibition.

## 9. Treatment

The genetic component of IgA glomerulopathy pathogenesis has been well demonstrated, yet until recently specific treatments for it have not been established, relying predominantly on supportive therapy with the potential addition of immunosuppressive therapy as an adjunct [4,49]. The overarching management of IgAN till now has consisted mainly of generalized tools used for the management of chronic kidney disease (CKD), and these were not IgA-specific; however, now the treatment regimen has evolved to include a combination of optimised chronic kidney disease therapies and treatments aimed at addressing defined components of the IgAN pathogenesis process [4].

Supportive management remains a core component of any IgAN treatment regimen and involves lifestyle modifications, blood pressure monitoring, maximal tolerated levels of renin–angiotensin system inhibition and sodium–glucose co-transporter-2 inhibition (SGLT-2) in eligible patients [4,60]. The selection of treatment for a patient should involve the consideration of such factors like patient’s risk of progression, treatment contraindications and whether the available evidence demonstrates only a reduction in proteinuria or a confirmed benefit on kidney-function decline [4].

The goal of treatment has two main components: the primary is to reduce the formation or renal effects of pathogenic IgA-containing immune complexes and the second is to limit the consequences of established nephron loss [4]. According to the KDIGO 2025 guideline, a nine-month course is recommended for targeted-release budesonide for patients at risk of progressive kidney-function loss; the guideline also recognises the role of sparsentan, where it is approved, as an alternative to conventional renin–angiotensin system inhibition rather than as an additional agent [4]. Mycophenolate mofetil may be considered as a glucocorticoid-sparing treatment in Chinese patients, whereas evidence remains insufficient to extend this recommendation to non-Chinese populations [4,61].

### 9.1. Approved IgAN-Directed Therapies

One of these new therapies which have recently been added into the possible treatment regimen for IgAN is targeted-release budesonide, which delivers budesonide to the distal ileum and targets mucosal immune processes which have been implicated in pathogenic IgA production [62,63]. It was tested in a randomised, placebo-controlled phase III trial, referred to as the “NefIgArd Trial,” in which 364 participants received a nine-month course of the agent or a placebo [62]. The study further included a 15-month off-treatment observational period [62]. It was noted that the treatment resulted in reduced proteinuria and produced a two-year benefit in eGFR (time-weighted mean change in eGFR over two years of −2.47 mL/min/1.73 m^2^ with targeted-release budesonide versus −7.52 mL/min/1.73 m^2^ with placebo), supporting its full approval by the FDA in December of 2023 [62,63].

Another agent which has been granted approval by the FDA is Sparsentan, a dual endothelin type A and angiotensin II type 1 receptor antagonist [64,65,66]. The agent’s phase III PROTECT trial compared sparsentan with the maximal titrated dose of irbesartan in over 404 patients over a time frame exceeding 110 weeks [64,65]. It was demonstrated that sparsentan led to a sustained reduction in proteinuria (UPCR reduction of 42.8% versus 4.4% at week 110) and benefit in regard to the chronic eGFR slope relative to irbesartan [64,65]. Since sparsentan blocks the angiotensin II type 1 receptor, it is not used as an adjunct but rather as a replacement for traditional angiotensin-converting enzyme inhibitor or angiotensin-receptor blocker therapy [4,66]. It was officially given FDA approval under the commercial name FILSPARI for IgA nephropathy, with full approval granted in September 2024 [66]. It is labelled with warnings regarding hepatotoxicity as well as embryo-foetal toxicity [66].

Atrasentan is another drug that has been approved for use in IgAN, which was granted accelerated FDA approval in April 2025 [67,68]. It is a selective endothelin type A receptor antagonist that reduces proteinuria in adults with primary IgAN who are at risk of rapid disease progression [67,68]. This aforementioned approval was based on a prespecified week-36 proteinuria analysis during the randomised, placebo-controlled phase III ALIGN trial, in which UPCR declined by 38.1% from baseline with atrasentan and by 3.1% with placebo [67]. At the time of accelerated approval, an effect on long-term kidney-function decline has not been established; therefore, the proteinuria result should be presented as a surrogate endpoint rather than consequential proof of nephroprotection [68].

Iptacopan is a medication that was granted accelerated FDA approval in 2024, but compared to the other previously mentioned agents, it acts on a different pathway [69,70]. It is an oral factor B inhibitor that is a selective suppressant of the alternative complement pathway [69]. The accelerated approval was granted due to the promising proteinuria reduction and was followed by traditional FDA approval in July of 2026, after the completion of the third phase of the APPLAUSE-IgAN trial [70]. The trial itself was a randomised, placebo-controlled trial which followed 477 patients over a treatment course of two years, where it was evidenced that the annualised total eGFR slopes were approximately −3.0 and −5.7 mL/min/1.73 m^2^/year, the former for iptacopan and the latter for the placebo [70]. Iptacopan distinguishes itself from complement agents that only have supportive evidence such as proteinuria or pharmacodynamic findings, yet iptacopan has confirmatory kidney-function evidence now available [70].

### 9.2. APRIL-TACI-Directed Therapies

We have previously discussed the APRIL-TACI axis in regard to its significance for IgAN; however, it also remains particularly relevant as a therapeutic frontier, as it is a link between human genetic evidence and therapeutic development [49,71]. An international GWAS which involved around 10,146 biopsy-confirmed IgAN cases and 28,751 controls identified risk loci implicating APRIL- and TACI-related signalling [49]. These findings support the value of this pathway as a potential treatment target but it does not establish that individual risk variants predict treatment response [49].

Sibeprenlimab is one such therapeutic, being a monoclonal antibody that selectively inhibits APRIL [71,72]. It received accelerated FDA approval in November 2025 to reduce proteinuria in adults with primary IgAN at risk of progression [72]. In the prespecified interim analysis of the placebo-controlled phase III VISIONARY trial, the 24 h UPCR decreased by 50.2% at nine months in the sibeprenlimab group and increased by 2.1% in the placebo group, corresponding to a 51.2% placebo-adjusted reduction [73]. Because accelerated approval was based on proteinuria, it had not been established at the time of approval whether sibeprenlimab slows long-term kidney-function decline; the published interim report likewise evaluated the nine-month proteinuria endpoint, with the annualised eGFR slope over 24 months specified as a key secondary endpoint to be reported at trial completion [72,73].

Another drug that functions on the APRIL–TACI axis is atacicept. Atacicept is a soluble TACI–Fc fusion protein that neutralises both APRIL and B-cell activating factor [74]. In the prespecified interim analysis of the randomised phase III ORIGIN 3 trial, atacicept produced a 41.8-percentage-point greater reduction in 24 h UPCR than placebo at week 36, which supported its accelerated FDA approval in July 2026 [74,75]. Because the published analysis was based on proteinuria at week 36, the initial regulatory decision should not be considered definitive proof of long-term nephroprotection [74,75].

### 9.3. Systemic Immunosuppression and Population-Specific Evidence

As discussed, immunosuppression is a key component in treating nephropathies; however, the specific genetic differences also require different treatment approaches. One example of the potential population disparity is evidence concerning systemic glucocorticoids and mycophenolate mofetil among clinical trial populations [4,61,76]. The MAIN trial demonstrated a benefit to the addition of mycophenolate mofetil as supportive therapy in 170 Chinese patients with progressive IgAN; it is important to stress that these differences found in a study conducted within one national population cannot ascertain that treatment response is genetically determined or able to be generalised to the Asian population as a whole [61]. Due to this, current KDIGO guidelines describe mycophenolate mofetil as a potential glucocorticoid-sparing option in Chinese patients while concluding that evidence remains insufficient in non-Chinese patients [4].

Furthermore, differences between systemic glucocorticoid trials may reflect study eligibility, baseline progression rate, level of kidney function, histological activity and chronicity, supportive care optimisation, glucocorticoid regimen, end-point definition, or statistical power [4,76,77]. The TESTING study did report benefits in its enrolled population—with the primary composite kidney outcome occurring in 7 of 121 reduced-dose methylprednisolone-treated participants versus 22 of 120 placebo-treated participants, alongside more frequent serious adverse events with methylprednisolone. However, its non-Chinese subgroup should not be characterised as predominantly Caucasian or European, and the study also did not collect sufficient genetic data to test a pharmacogenomic interaction [76]. STOP-IgAN, a study conducted in Germany, noted no durable kidney-function improvement from adding immunosuppression to intensive supportive care. Yet, the distinctions between these trials do not independently demonstrate any ancestry-dependent efficacy [77].

Due to the above, population-specific evidence should be reported according to where and on which patients the trials were conducted, without prematurely assuming any observed changes should be attributed to genetic ancestry unless a prospectively specified genotype-by-treatment interaction has been demonstrated.

### 9.4. Investigational Complement and Nucleic Acid Therapies

There is true pioneering potential for IgAN treatments that address the disease on a molecular level. These include the gene addition approaches such as PS-002 [78] and a range of new and innovative nucleic acid-based therapies such as Sefaxersen, Cemdisiran, and ARO-C3 [79,80,81] in particular. These approaches are molecularly targeted but do not alter a patient’s inherited genome and should not be described as gene therapies.

Sefaxersen, otherwise known as IONIS-FB-LRx during development [79], is a GalNAc-conjugated 2′-O-methoxyethyl (2′-MOE) antisense oligonucleotide that targets hepatic CFB, reducing complement factor B mRNA [79]. Since an overactive complement pathway has been implicated in research regarding the pathophysiology of IgAN development [79], the suppression of it should result in a clinically better presentation for patients. In fact, Sefaxersen was recently the subject of an open-label phase II study in which 23 IgAN patients were recruited to test the initial safety and potential efficacy of the drug [79]. All patients with IgAN completed the study, with an acceptable safety profile of the drug; one treatment-emergent serious adverse event occurred and was judged unrelated to treatment, and three participants developed transient, reversible alanine aminotransferase elevations of approximately three to five times the upper limit of normal that resolved without treatment discontinuation [79]. The trial also demonstrated a clinically significant reduction in CFB and improvements in the levels of proteinuria: participants received sefaxersen 70 mg subcutaneously for 24 weeks, and at week 29, 24 h urinary protein excretion had decreased by 43%, from a geometric mean of 2.5 to 1.4 g/day, with similar reductions in UPCR and UACR and stable mean eGFR [79]. Sefaxersen also reduced plasma factor B and Bb, urinary Ba and serum alternative-pathway activity without materially suppressing classical-pathway activity, supporting the theory that targeting the complement pathway can result in a reduction in the clinical severity of disease progression [79]. The trial also established the regimen that is currently the basis for the Phase 3 trial, “IMAgINATION”—wherein a dose of 70 mg of sefaxersen is administered subcutaneously on the 1st, 15th and 29th day of the study, with a follow-up dose every 4 weeks through week 105—to ensure durability of its effect [82]. The single-arm design, small sample and short controlled observation period mean the second-phase findings are insufficient evidence of disease progression reduction [79].

Cemdisiran is a further complement inhibitor that has completed Phase II testing [80]. It, in turn, is a double-stranded small interfering RNA that, through its covalent linkage to N-acetylgalactosamine, enables the downregulation of hepatic C5 mRNA [80], which is the final protein in the complement cascade prior to the formation of the membrane attack complex (MAC), which is a great contributor to cell injury and tissue inflammation in the kidneys [80]. The inhibition of this complement marker should decrease the generation of C5 and, by extension, MAC, extending the period before disease progression [80]. The phase I trials did demonstrate a swift and robust response of the drug on its intended target, and this was further underscored by the randomised, double-blind phase II trial, which reported a clinically meaningful magnitude of proteinuria reduction with cemdisiran, a surrogate end-point for monitoring kidney damage; the study was descriptive, conducted over 36 weeks and not designed for formal hypothesis testing [80]. The second phase consisted of 31 patients, with 22 receiving cemdisiran 600 mg and 9 in the placebo control group; cemdisiran was administered on a monthly basis subcutaneously [80]. In those patients, at 32 weeks there was a placebo-adjusted decrease in 24 h urine protein-to-creatinine ratio (UPCR) of 37.4%, with spot UPCR down by 45.8% for the placebo-adjusted geometric mean ratio change from baseline [80]. This was accompanied by a mean change in C5 at week 32 of −98.7% of serum C5; in contrast, in the placebo group, C5 increased by 25.2% [80]. This signifies the strong inhibition of anaphylatoxin C5a and MAC production [80].

Whilst these results are greatly encouraging, it is important to take into consideration the various adverse events that were noted during the trial, including one death; however, this death consisted of a cardio-respiratory collapse and was noted as a complication following the patient undergoing bypass surgery and was judged unrelated to cemdisiran [80]. While 41% of cemdisiran-treated participants experienced injection-site reactions, and the majority of other events were mild to moderate and transient, two patients did experience elevations in alanine aminotransferase (ALT) and aspartate aminotransferase (AST), which did self-resolve with no alteration of treatment or drug dose [80]; however, the sample size of the trial was small and requires a large participant pool to truly evaluate any further drug-related treatment complications. Cemdisiran monotherapy in IgAN has not subsequently progressed to a registered Phase III programme [77,80].

ARO-C3, an investigational RNA interference therapeutic that targets the hepatic expression of complement C3 production, has recently announced the completion of its Phase I/IIa study and presented its “selected and top-line” results that offer an additional beacon of hope for innovative RNA-modifying treatments for IgAN; these findings derive from sponsor-reported top-line data and conference materials rather than a complete peer-reviewed clinical publication and should be identified accordingly [81]. The study recruited 14 adults with IgAN, who received 400 mg of ARO-C3 subcutaneously on days 1, 29 and 113 of the study period, with follow-up on day 169 [81]. The baseline mean eGFR of IgAN patients was 80.6 mL/min/1.73 m^2^, with a spot UPCR of 2051 mg/g; the trial also began with 10 of the 14 patients testing 1+ or more on dipstick tests for hematuria. It concluded with only 3 patients persistently testing positive for hematuria by week 24 [81]. Furthermore, it also revealed a maximum mean reduction in serum C3 of approximately 89%, with a sustained decrease of over 87% within the study timeframe [81]. Complement activity, which was assessed through the 50% alternative pathway haemolytic activity (AH50) assay, the 50% total haemolytic complement activity (CH50) assay, and the Wieslab alternative pathway assay, all illustrated reductions of 85.2%, 63.8%, and 98%, respectively, which were sustained at more than 76%, 61%, and 87% decreases [81]. Moreover, the mean UPCR denoted a drop of approximately 41% by day 169, with the maintenance of stable renal function [81]. All adverse events that occurred throughout the duration of the trial were mild or moderate in severity, with no severe events being noted. The most commonly reported side effects included headaches, a cough, and nasopharyngitis [81]. While the trial was taking place, the patients did not present at increased risk of encapsulated organism infections, nor were there any participant deaths or discontinuations [81]. Yet, the sample size of the study was quite limited (14 IgAN patients), and generalized conclusions regarding its long-term safety profile and related adverse events would first need to be monitored in a larger patient population; these uncontrolled observations therefore do not establish comparative efficacy or long-term nephroprotection. Currently, no further study plans for ARO-C3 have been officially announced [81].

A further potential treatment is the long-acting monoclonal antibody ravulizumab, which inhibits complement C5 [83]. In a pre-specified interim analysis of the phase III I CAN trial, sponsor-reported results showed that ravulizumab decreased 24 h UPCR by 46.6% from baseline at week 34, compared to a 5.6% drop in the placebo group [83]. This corresponded to the placebo-adjusted effect of 43.4% [83]. The FDA accepted a supplemental biologics licence application and granted priority review in June 2026, with an anticipated decision date in the fourth quarter of 2026; at the evidence cutoff for this review, ravulizumab was not FDA-approved for IgAN [83]. The week-106 eGFR endpoint still remains pending, and the interim results were available primarily through a conference presentation and sponsor disclosure rather than a complete peer-reviewed Phase III publication [83].

### 9.5. Gene Addition and Editing Approaches

PS-002 is an investigational gene addition therapy under development and is currently set for Phase I/II clinical trials [78]. This phase is planned for the establishment of safety parameters and efficacy biomarkers that will set the basis for the dose for inclusion in the next phase of testing [78]. PS-002 itself is an adeno-associated virus (AAV)-vector-mediated gene addition therapy being investigated for IgAN that targets the complement pathway [78,84]. The adeno-associated virus serotype 9 (AAV-9) contains the human complement factor I gene (*CFI*), targeting podocytes [84]. CFI is a natural negative regulator of the alternative pathway, and the therapy aims to provide long-term treatment after single-dose administration [84]. It should be described as a gene addition therapy rather than a correction of an inherited IgAN-causing mutation, because IgAN is generally a complex polygenic and immune-mediated disease [49,78].

Prior to human trials, it has been tested in both murine and pig models, where it demonstrated a decrease in proteinuria, mesangial C3 deposition, inflammation, and casts, as well as fibrosis [84]. Post-administration in three Göttingen mini pigs, PS-002 demonstrated stable gene expression, glomerulus-wide up to 56 days, and no reported adverse events in either murine or pig models [84]. It also demonstrated blunting of kidney scarring and decreasing glomerulosclerosis [84]. These findings have been presented through conference and company materials rather than a complete peer-reviewed disease-specific publication and should therefore be labelled explicitly as preliminary preclinical evidence [84]. The first in human trials will be open label phase I/II trials, which have a target enrollment of 32 adults, and began recruiting in 2026. They will involve a single-dose administration with patient monitoring up to one year, with a long-term follow-up at year five post-administration [78]. The study has one centre dropped out; 10 locations as of now encompassing both the EU—where it received orphan drug designation in April 2025—and the USA, where it received FDA IND clearance to begin clinical testing in July of 2025; IND clearance permits the initiation of clinical testing and is not equivalent to FDA marketing approval [78]. Whilst there has been an influx of promising therapies in development for IgAN, a proposed benefit of PS-002 is its one-time administration that would lead to significantly prolonged expression and podocyte specificity; these advantages remain hypotheses to be evaluated in humans rather than established clinical benefits [78]. Although the use of AAV vectors has been a significant step forward, it is important to note valid concerns about their widespread usage. The related concerns regarding AAV vector use stem from the adverse events that have been noted in association with their implementation in other diseases [85].

AAV vectors themselves are single-stranded (ssDNA) DNA Parvoviridae, which are packed into non-enveloped icosahedral capsids of 25 nm diameter [85,86]. These are used to express genes of interest in cell and animal models. They have a growing range of applications due to their non-pathogenic nature and their predominantly episomal persistence; low-frequency genomic integration cannot be excluded, so they should not be described as categorically non-integrating [85,86]. While several concerns about the efficacy of their use for kidney transduction due to their wide variability have been emphasised, a 2024 comparison of 47 capsids showed that systemically administered AAV-9 achieved greater renal tubular and podocyte transduction than other capsids when the route of administration was via intravenous injection (IV), whereas locally administered capsids such as AAV-KP1 more efficiently transduced proximal tubules following renal vein (RV) or renal pelvis (RP) delivery [87]. This demonstrates that capsid selection, promoter, route of administration, disease state and species are of key significance in the development of new gene therapies, as the choice underscores their potential efficacy and role as one-time application curative treatments, while no particular capsid has been established as optimal in humans [87]. AAVs have been applied in several clinical conditions for gene therapy trials and showcased potential complications of their use; systemically delivered, high-dose AAV therapies, including AAV9-based treatment in spinal muscular atrophy, have been associated with complement activation and thrombotic microangiopathy [85]. In the context of renal disease therapy, a localized administration approach could enable the use of lower doses for treatment and effectively reduce systemic exposure and the associated safety risks, although a reduction in clinical toxicity has not yet been demonstrated for PS-002 [78,85,87]. Another potential obstacle to their implementation is pre-existing immunity, which can reduce vector efficacy, limit eligibility and lead to serious complications with re-dosing, thus making this generally avoided [86]. Therefore, for the success of curative AAV therapy in IgAN, there must be stable and sustained transgene expression for long-term success. Encouragingly, in haemophilia AAV hepatic gene trials, transgene expression has persisted for more than ten years in some haemophilia B studies and for approximately five years in haemophilia A, although substantial interindividual variability and declining expression have been reported [88]. This is promising, especially since podocytes have a much lower turnover as compared to hepatocytes and thus a single administration could lead to prolonged expression, though this does not establish that one administration will be lifelong or curative [78,88].

A commercial research concept has proposed CRISPR-Cas-mediated kidney editing using viral, nanoparticle, in vivo and ex vivo delivery strategies as a next potential frontier for the treatment. Crucially, these concepts cannot currently be classified as IgAN therapies in development due to the absence of peer-reviewed disease-specific preclinical evidence or a registered clinical program/study design.

### 9.6. Target-Specific Safety Considerations

Systemic glucocorticoids increase serious infection and other dose-dependent toxicities [4,76]. This favors reduced-dose regimens, prophylaxis when indicated, and careful patient selection [4,76]. Targeted-release budesonide reduces systemic exposure relative to conventional corticosteroids but still produces glucocorticoid-related adverse effects—including peripheral oedema, hypertension, acne and weight gain—and should not be described as exclusively local [62,63].

Endothelin-pathway agents require monitoring for fluid retention, hypotension, and hepatic effects, together with strict pregnancy precautions where mandated [66,68]. Sparsentan carries boxed warnings for hepatotoxicity and embryo–fetal toxicity and replaces ACE-inhibitor or angiotensin-receptor-blocker therapy; it must not be co-administered with another angiotensin-receptor blocker, endothelin-receptor antagonist or aliskiren [66]. Atrasentan lacks angiotensin-receptor blockade and therefore has a different place in combination treatment, having been evaluated on top of background renin–angiotensin system inhibition [67,68].

The infection risk of complement inhibition depends on the level and pathway inhibited and should not be generalized from eculizumab to every complement-directed agent; the magnitude and pattern of risk vary with the complement target, the degree of inhibition and the treated population [70]. Thereby, it stands to reason that other drugs impacting the inhibition of the complement pathway may generate similar safety concerns; the exclusion of this risk requires rigorous large-scale testing, with long-term follow-up. Proximal and terminal complement blockade can impair defence against encapsulated organisms, particularly Neisseria meningitidis, Streptococcus pneumoniae, and Haemophilus influenzae [70]. Agent-specific prescribing information, vaccination requirements, antimicrobial prophylaxis, and REMS obligations should be reported; for example, iptacopan carries a boxed warning for serious and life-threatening infections caused by encapsulated bacteria and REMS requirements [70].

APRIL and BAFF/APRIL inhibition can alter B-cell and plasma-cell biology, immunoglobulin concentrations, vaccine responses, and infection risk [72,74]. For sibeprenlimab, the most common labelled adverse reactions are upper respiratory tract infection and injection-site erythema; the FDA review identified infection and interference with vaccine responses as mechanistic risks but did not require a REMS [72]. For atacicept, the most common labelled adverse reactions include upper respiratory tract infection and injection-site reactions, and the label advises monitoring for infection and avoiding live vaccines around treatment; in the prespecified phase III interim analysis, adverse events occurred in 59.3% of atacicept-treated patients and 50.0% of placebo-treated patients and were mostly mild or moderate in severity [74,75].

### 9.7. Biomarker and Outcome Limitations

Proteinuria is a clinically meaningful and widely accepted surrogate in IgAN, and sustained lower proteinuria is associated with better kidney outcomes [4,89]. Nevertheless, proteinuria reduction does not by itself prove long-term nephroprotection for every mechanism [4]. Evidence should be stratified by: pharmacodynamic target engagement; proteinuria reduction; eGFR-slope benefit; or reduction in hard kidney outcomes. Accelerated approval based on proteinuria should not be described as equivalent to traditional approval based on confirmatory kidney-function evidence.

No genetic, serum, urinary, or biopsy complement biomarker is currently validated to select a particular complement inhibitor in routine IgAN care or to predict its incremental benefit [4]. KDIGO states that no validated prognostic serum or urine biomarkers are available beyond eGFR and proteinuria, while histology and emerging complement assays may refine future risk stratification but do not yet establish treatment eligibility [4]. Complement-directed therapies should therefore be used according to approved indications and trial eligibility, not restricted to patients classified as “complement activated” [4].

Similarly, population-specific therapeutic evidence must not be equated with pharmacogenomic evidence. Geographic or ethnic differences in trial outcomes can arise from clinical eligibility, baseline risk, pathology, background care, treatment regimen, adherence, endpoint definitions, or chance [4,61,76,77]. A claim that ancestry or genotype predicts response requires a prospectively specified and adequately powered interaction analysis. Table 3 presents the approved and investigational therapies in IgaN.

## 10. Limitations

This review has a few main limitations concerning the genetic evidence. First, association does not identify the causal gene. Apart from *CFHR3,1Δ*, the missense variant rs3803800 in *TNFSF13* and pathogenic *COL4A3/COL4A4/COL4A5* variants, the assignment of signals to genes rests on proximity to the lead variant and on biological plausibility. What is more, several of the candidate-gene findings discussed here come from single, modestly sized, single-ancestry cohorts that await replication. Finally, the allocation of loci to individual hits remains hypothetical. Most loci plausibly act at more than one step, the third hit has no identified genetic determinant, and the fact that a gene participates in an immune process does not tell us when in the sequence it matters. A further limitation concerns severity. Genetic studies in IgAN have almost invariably used disease susceptibility as the outcome, and few report histological grading according to the Oxford MEST-C classification or hard kidney endpoints. Statements about genetic modulation of disease severity therefore rest on a much thinner evidence base than statements about susceptibility.

## 11. Conclusions

The evidence reviewed here supports the view that IgAN is a polygenic disease in which inherited variation contributes to both susceptibility and clinical course.

What has changed in IgAN is less the volume of genetic data than what that data is used for. When susceptibility loci are mapped onto the four-hit sequence, a stepwise architecture emerges. The alternative complement pathway has been the first of these nodes to become therapeutically accessible, because it combines a strong association with an identified causal allele, a pathway measurable in blood and inhibitors already in clinical development. It allows IgAN to move from a disease managed with non-specific care to one with a pipeline of mechanism-directed agents.

Early-phase results have been encouraging, with substantial and sustained reductions in complement activity accompanied by clinically meaningful falls in proteinuria.

On the other hand, there are several problems that temper the optimism. Trials to date remain small, short and surrogate-endpoint driven. Sustained complement inhibition also carries an infectious risk that differs by target, and no biomarker is yet validated to identify the patients most likely to benefit. African and admixed populations remain underrepresented, and most associated variants are functionally unvalidated. Closing these gaps is the most plausible route from genetic architecture to individualized treatment.

## Figures and Tables

**Table 1:** Evidence for a genetic contribution to IgAN.

Evidence	Key Finding
Geographic/ethnic gradient	IgAN frequency highest in East Asia (~45% of biopsies), intermediate in Europe (~25%), lowest in Africa (<5%)
Geospatial genetic risk score	Seven-SNP risk score tracks the prevalence gradient (highest in East Asians, lowest in Africans) and follows ancestry rather than environment in diaspora populations
Familial aggregation & inheritance	Familial clustering, predominantly autosomal dominant with variable penetrance; commoner in European families and associated with worse outcomes
Sex difference	Incidence ~5× higher in men than women; male sex predicts poor renal outcome (difference attenuated in familial cases)
Heritability of Gd-IgA1	Serum Gd-IgA1 highly heritable (~80% in twins; 40–80% in family studies)
Epigenetic regulation	MZ twins discordant for IgAN differ in DNA methylation at immune genes, independent of genotype

Abbreviations: Gd-IgA1, galactose-deficient IgA1; IgAN, IgA nephropathy; MZ, monozygotic; SNP, single-nucleotide polymorphism.

**Table 2:** Genetic contributions mapped onto the four-hit hypothesis.

Hit (Step)	Implicated Genes/Loci (Key Variants)	Genetic Note
First hit—aberrant IgA1 O-glycosylation (Gd-IgA1 increase)	*C1GALT1* (rs13226913), *C1GALT1C1*/Cosmc (rs5910940), *ST6GALNAC2* (promoter variants), *GALNT12*; *TLR1* (rs4833095/rs5743557)	Glycosylation-enzyme genes set Gd-IgA1 levels; reinforced by epigenetic control (miR-148b → *C1GALT1*, let-7b → *GALNT2*, *C1GALT1* promoter methylation)
Second hit—anti-glycan IgG autoantibodies	MHC class II (6p21); *TNFSF13/APRIL* (17p13, rs3803800), *TNFRSF13B/TACI* (17p11.2)	MHC alleles permit T-cell help; *TNFSF13* and *TNFRSF13B* are independent risk loci encoding a ligand–receptor pair. The high-affinity IgG substitution itself is somatic, not inherited
Third hit—circulating immune-complex formation	No defined genetic determinants	Largely a physicochemical step with no defined independent genetic determinants
Fourth hit—mesangial deposition & complement-mediated injury	*CFHR1/CFHR3* (1q32), *CFH/CFHR1-CFHR3* (rs6677604-A/*CFHR3,1Δ* deletion, protective; rare *CFH* variants), *CFB* (rs4151657)	Best characterised genetic modulation, via the alternative complement pathway; *CFHR3,1Δ* frequency differs by population;
Across all steps—multi-ancestry GWAS	30 risk loci	Risk clusters in four processes: antigen presentation (MHC), mucosal/intestinal IgA immunity (*TNFSF13, DEFA*, *LIF*/*OSM*), complement (*CFH/CFHR*), NF-κB innate signaling (*RELA, REL, LYN*)

Gene names indicate the candidate prioritised in the source publication; unless stated otherwise, this assignment is based on proximity to the lead variant. The strength of the evidence supporting each assignment is discussed in Section 8.4. Abbreviations: APRIL, a proliferation-inducing ligand; *C1GALT1*, core 1 β1,3-galactosyltransferase 1; *C1GALT1C1*, C1GALT1-specific chaperone 1; *CFB*, complement factor B gene; *CFH*, complement factor H gene; CFHR1/CFHR3, complement factor H-related protein 1/3; *CFHR3,1Δ*, 84 kb deletion encompassing *CFHR3* and *CFHR1*; Cosmc, core 1 β3-Gal-T-specific molecular chaperone; *DEFA*, α-defensin gene; *GALNT2/GALNT12*, polypeptide N-acetylgalactosaminyltransferase 2/12; Gd-IgA1, galactose-deficient IgA1; GWAS, genome-wide association study; IgA1, immunoglobulin A1; IgAN, IgA nephropathy; IgG, immunoglobulin G; let-7b, lethal-7b microRNA; *LIF*, leukaemia inhibitory factor; *LYN*, *LYN* proto-oncogene, Src-family tyrosine kinase; MHC, major histocompatibility complex; miR-148b, microRNA-148b; mRNA, messenger RNA; NF-κB, nuclear factor κB; *OSM*, oncostatin M; *REL/RELA*, *REL* proto-oncogene/*RELA* proto-oncogene, NF-κB subunit; rs, reference SNP cluster identifier; *ST6GALNAC2*, ST6 N-acetylgalactosaminide α-2,6-sialyltransferase 2; TACI, transmembrane activator and CAML interactor; *TLR1*, Toll-like receptor 1; *TNFRSF13B*, TNF receptor superfamily member 13B; *TNFSF13*, TNF superfamily member 13.

**Table 3:** Approved and investigational therapies targeting the molecular pathogenesis of IgA nephropathy.

Agent	Target and Mechanism	Regulatory Status	Trial (Phase, *n*)	Comparator and Duration	Key Efficacy Result	Genetic Support for the Target
**Approved IgAN-directed therapies**						
Targeted-release budesonide	Delivery of budesonide to the distal ileum; mucosal immune processes implicated in pathogenic IgA production	Full FDA approval, December 2023	NefIgArd, phase III, *n* = 364	Placebo; 9-month treatment plus 15-month off-treatment follow-up	Two-year time-weighted mean change in eGFR −2.47 vs. −7.52 mL/min/1.73 m^2^ (each from baseline); proteinuria reduced	Pathway level: mucosal IgA loci (*TNFSF13*, *DEFA*, *LIF*/*OSM*) are genome-wide significant
Sparsentan	Dual endothelin type A and angiotensin II type 1 receptor antagonist	Accelerated FDA approval February 2023 (proteinuria); traditional approval September 2024 (kidney-function decline)	PROTECT, phase III, *n* = 404	Maximally titrated irbesartan; >110 weeks	UPCR −42.8% vs. −4.4% at week 110 (each from baseline); benefit in chronic eGFR slope	None: no IgAN risk locus at the endothelin or angiotensin pathway
Atrasentan	Selective endothelin type A receptor antagonist	Accelerated FDA approval, April 2025	ALIGN, phase III, *n* = 404 randomised (340 main stratum, 64 SGLT2i stratum); prespecified interim analysis of the first 270 patients of the main stratum	Placebo; 132 weeks of treatment, primary endpoint at week 36	UPCR −38.1% vs. −3.1% from baseline; between-group difference −36.1 percentage points (95% CI −44.6 to −26.4); effect on kidney-function decline not established at approval	None
Iptacopan	Oral factor B inhibitor; selective inhibition of the alternative complement pathway	Accelerated FDA approval 2024; traditional approval July 2026	APPLAUSE-IgAN, phase III, *n* = 477	Placebo; 2 years	Annualised total eGFR slope −3.0 vs. −5.7 mL/min/1.73 m^2^/year	Indirect: *CFB* supported by candidate-gene studies only, but within the pathway containing *CFH*/*CFHR*, the strongest non-MHC locus
**APRIL–TACI-directed therapies**						
Sibeprenlimab	Monoclonal antibody; selective inhibition of APRIL	Accelerated FDA approval, November 2025	VISIONARY, phase III, *n* = 510 randomised (259 sibeprenlimab, 251 placebo); prespecified interim analysis of the first 320 patients	Placebo; 9 months	24 h UPCR −50.2% vs. +2.1% from baseline; placebo-adjusted −51.2% (96.5% CI 42.9–58.2); two-year eGFR findings announced subsequently [73]	Target level: *TNFSF13* is genome-wide significant and carries the coding variant rs3803800
Atacicept	Soluble TACI-Fc fusion protein; neutralises both APRIL and BAFF	Accelerated FDA approval, July 2026	ORIGIN 3, phase III, *n* = 431 randomised; prespecified interim analysis of 203 patients (106 atacicept, 97 placebo)	Placebo; week 36	24 h UPCR −45.7% vs. −6.8% from baseline; between-group difference 41.8 percentage points (95% CI 28.9–52.3) [75]	Target level: *TNFSF13* and *TNFRSF13B* are independent genome-wide significant loci
**Investigational complement-directed and nucleic acid therapies**						
Sefaxersen (IONIS-FB-LRx)	GalNAc-conjugated 2′-MOE antisense oligonucleotide; reduces hepatic complement factor B mRNA	Investigational; phase III IMAgINATION ongoing	Open-label phase II, *n* = 23	Single-arm, no comparator; 24 weeks of dosing, assessment at week 29	24 h urinary protein −43% from baseline (2.5 to 1.4 g/day); plasma factor B and alternative-pathway activity reduced	Indirect: *CFB*, candidate-gene evidence only
Cemdisiran	GalNAc-conjugated siRNA; downregulates hepatic C5 mRNA	Investigational; phase II completed	Phase II, *n* = 31 (22 cemdisiran, 9 placebo)	Placebo; 36 weeks, endpoint at week 32	24 h UPCR −37.4%, placebo-adjusted; serum C5 −98.7% vs. +25.2%	None at *C5*; rationale is pathway-based
ARO-C3	RNA interference therapeutic; targets hepatic complement C3 expression	Investigational; phase I/IIa completed (sponsor-reported topline data)	Phase I/IIa, *n* = 14	Single-arm, no comparator; assessment at day 169	UPCR ≈ −41% from baseline; serum C3 ≈ −89%	None at *C3*; rationale is pathway-based
Ravulizumab	Long-acting monoclonal antibody; inhibits complement C5	Not approved for IgAN; sBLA under FDA priority review, decision expected Q4 2026	I CAN, phase III, prespecified interim analysis (sponsor-reported)	Placebo; week 34	24 h UPCR −46.6% vs. −5.6% from baseline; placebo-adjusted −43.4%; week-106 eGFR endpoint pending	None at *C5*; rationale is pathway-based
**Gene addition approaches**						
PS-002	AAV-9 vector delivering the human complement factor I gene (*CFI*) to podocytes	Investigational; FDA IND clearance only, phase I/II planned (not an approval)	Preclinical: murine models and three Göttingen mini pigs	No human data	Reduced proteinuria, mesangial C3 deposition, inflammation and fibrosis in animals; conference and company materials rather than peer-reviewed disease-specific publication	None at *CFI*; rationale is pathway-based

Unless stated otherwise, efficacy results are changes from baseline within each treatment group; placebo-adjusted values are identified as such. Accelerated approval based on proteinuria is not equivalent to traditional approval supported by confirmatory kidney-function evidence. Sponsor-reported and conference data are identified where they have not been published in full peer-reviewed form. The column on genetic support indicates whether the drug target itself is an established IgA nephropathy risk locus (target level), whether it lies within a pathway implicated by such a locus (pathway level or indirect), or whether no genetic association supports it; the evidential standard is the *CFH*/*CFHR* locus, discussed in Section 8.4. Abbreviations: 2′-MOE, 2′-O-methoxyethyl; AAV-9, adeno-associated virus serotype 9; APRIL, a proliferation-inducing ligand; BAFF, B-cell activating factor; C3, complement component 3; C5, complement component 5; eGFR, estimated glomerular filtration rate; FDA, US Food and Drug Administration; GalNAc, N-acetylgalactosamine; IgA, immunoglobulin A; IgAN, IgA nephropathy; IND, investigational new drug; MHC, major histocompatibility complex; RAS, renin–angiotensin system; sBLA, supplemental biologics licence application; siRNA, small interfering RNA; TACI, transmembrane activator and CAML interactor; UPCR, urine protein-to-creatinine ratio.

## Data Availability

No new data were created or analyzed in this study. Data sharing is not applicable to this article.

## Abbreviations

The following abbreviations are used in this manuscript:

2′-MOE	2′-O-methoxyethyl
AAV	Adeno-associated virus
AAV9	Adeno-associated virus serotype 9
ACE	Angiotensin-converting enzyme
AH50	50% alternative pathway haemolytic activity
ALT	Alanine aminotransferase
AP	Alternative pathway
APRIL	A proliferation-inducing ligand
ARB	Angiotensin-receptor blocker
AST	Aspartate aminotransferase
Ba, Bb	Activation fragments of complement factor B
BAFF	B-cell activating factor
C1GalT1	Core 1 β1,3-galactosyltransferase (protein)
C3	Complement component 3
C3b	Activated fragment of complement component 3
C5	Complement component 5
C5a	Anaphylatoxin fragment of complement component 5
CD4	Cluster of differentiation 4
CFHR3,1Δ	84 kb deletion encompassing CFHR3 and CFHR1
CH50	50% total haemolytic complement activity
CKD	Chronic kidney disease
Cosmc	Core 1 β3-Gal-T-specific molecular chaperone
CRISPR-Cas	Clustered regularly interspaced short palindromic repeats and CRISPR-associated protein
DNA	Deoxyribonucleic acid
eGFR	Estimated glomerular filtration rate
EMA	European Medicines Agency
ERA	Endothelin type A receptor antagonist
FDA	US Food and Drug Administration
FH	Complement factor H (protein)
GalNAc	N-acetylgalactosamine
GBM	Glomerular basement membrane
Gd-IgA1	Galactose-deficient IgA1
GWAS	Genome-wide association study
HLA	Human leukocyte antigen
IgA	Immunoglobulin A
IgA1	Immunoglobulin A1
IgAN	IgA nephropathy
IgG	Immunoglobulin G
IL-6	Interleukin 6
IL-18	Interleukin 18
IND	Investigational New Drug
IV	Intravenous
kDa	Kilodalton
KDIGO	Kidney Disease: Improving Global Outcomes
let-7b	Lethal-7b microRNA
MAC	Membrane attack complex
MAPK/ERK1/2	Mitogen-activated protein kinase/extracellular signal-regulated kinase 1/2
MHC	Major histocompatibility complex
miR-148b	MicroRNA-148b
miRNA	MicroRNA
mRNA	Messenger RNA
MZ	Monozygotic
NF-κB	Nuclear factor κB
rAAV	Recombinant adeno-associated virus
RAS	Renin–angiotensin system
REMS	Risk Evaluation and Mitigation Strategy
RNA	Ribonucleic acid
RP	Renal pelvis
RV	Renal vein
s.c.	Subcutaneous
SGLT-2	Sodium–glucose co-transporter 2
siRNA	Small interfering RNA
SNP	Single-nucleotide polymorphism
ST6GalNAc-II	ST6 N-acetylgalactosaminide α-2,6-sialyltransferase 2 (protein)
ssDNA	Single-stranded DNA
TACI	Transmembrane activator and CAML interactor
tRNA	Transfer RNA
UPCR	Urine protein-to-creatinine ratio
UTR	Untranslated region
Gene symbols	
*ANKRD16*	Ankyrin repeat domain 16
*C1GALT1*	Core 1 β1,3-galactosyltransferase 1
*C1GALT1C1*	C1GALT1-specific chaperone 1
*CCR6*	C-C motif chemokine receptor 6
*CFB*	Complement factor B
*CFH*	Complement factor H
*CFHR1, CFHR3*	Complement factor H-related protein 1, 3
*CFI*	Complement factor I
*COL4A3, COL4A4, COL4A5*	Collagen type IV α3, α4, α5 chain
*DEFA*	α-defensin
*FBXL21*	F-box and leucine-rich repeat protein 21
*GALNT2, GALNT12*	Polypeptide N-acetylgalactosaminyltransferase 2, 12
*IL1A, IL1B, IL1RN, IL10*	Interleukin 1α, interleukin 1β, interleukin 1 receptor antagonist, interleukin 10
*LYN*	LYN proto-oncogene, Src-family tyrosine kinase
*LIF*	Leukaemia inhibitory factor
*OSM*	oncostatin M
*PKD1L3*	polycystin 1-like 3
*REL, RELA*	REL proto-oncogene/RELA proto-oncogene, NF-κB subunit
*SPRY2*	Sprouty RTK signalling antagonist 2
*ST6GAL1*	β-galactoside α-2,6-sialyltransferase 1
*ST6GALNAC2*	ST6 N-acetylgalactosaminide α-2,6-sialyltransferase 2
*STAT3*	Signal transducer and activator of transcription 3
*TLR1, TLR2*	Toll-like receptor 1, 2
*TNFRSF13B*	TNF receptor superfamily member 13B
*TNFSF13*	TNF superfamily member 13
*VEGFA*	Vascular endothelial growth factor A
